# Supplementary material for: Plasmon-driven chemical transformation of a secondary amide probed by surface enhanced Raman scattering
Source: Commun Chem. 2024 Aug 27;7:188. doi: 10.1038/s42004-024-01276-2 (PMC11347659; doi:10.1038/s42004-024-01276-2)
Supplement: Supplementary file 1 — Supporting Information [file 42004_2024_1276_MOESM1_ESM.pdf]

Supplementary Information

Plasmon-Driven Chemical Transformation of a Secondary Amide Probed by Surface Enhanced Raman Scattering

Anushree Dutta[a,c], Milan Ončák[b], Farhad Izadi[b], Eugene Arthur-Baidoo[b], João Ameixa [a], Stephan Denifl[b], Ilko Bald[a,d]

[a] *Institute of Chemistry, University of Potsdam, Karl-Liebknecht-Str. 24-25, 14476, Potsdam, Germany.*

[b]*Institut für Ionenphysik und Angewandte Physik, Universität Innsbruck, Technikerstraße 25, 6020 Innsbruck, Austria*

[c]*Department of Chemistry and Applied Biosciences, ETH Zurich, Zurich CH-8093, Switzerland*

[d]*Dynamics of Molecules and Clusters Department, J. Heyrovský Institute of Physical Chemistry of the CAS, Dolejškova 3, Prague, 18223, Czech Republic*

*correspondence: bald@uni-potsdam.de

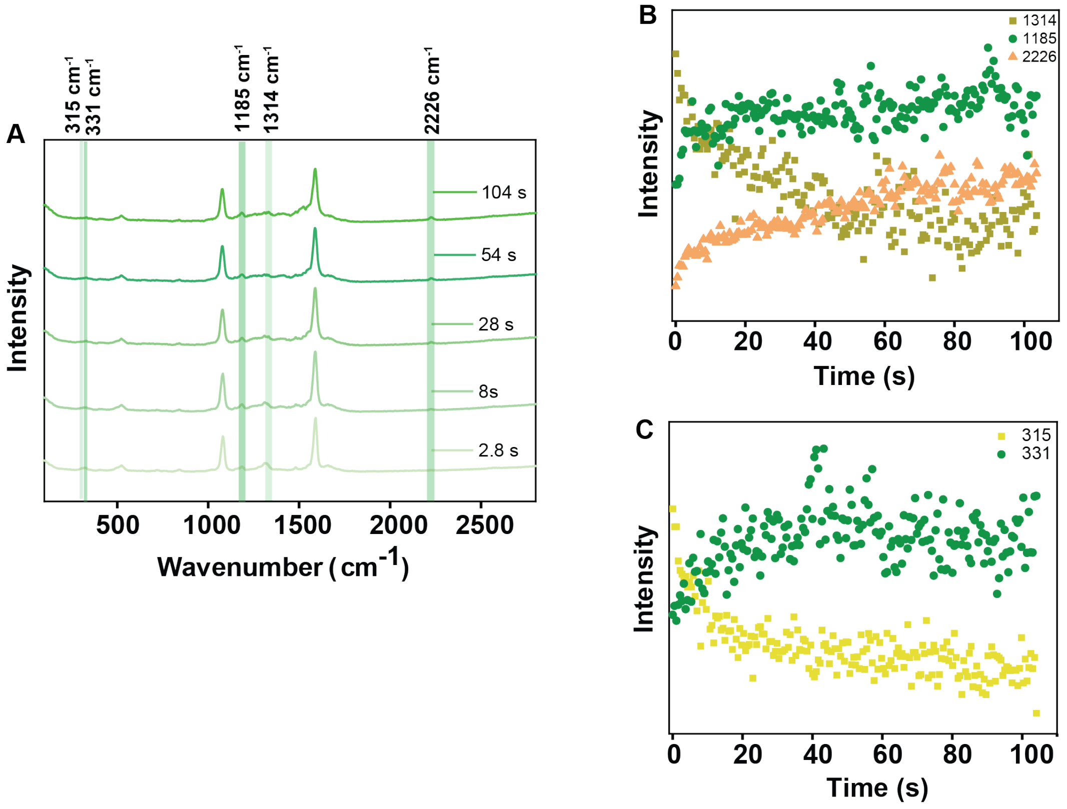

Supplementary Figure 1. Time evolution of the transformation of a secondary amide on Au aggregates tracked by SERS with 633 nm laser: Representative SERS spectra obtained at different time intervals of the chemical transformation of NMSB on Au-aggregates under 633 nm laser excitation are shown in (A). The time trajectories of the peaks at (B) 1314 cm^{-1}, 1185 $cm^{-1,}$ and 2226 cm^{-1} correspond to the loss of the amide III band, the appearance of the peak due to primary amide and aromatic nitrile, respectively, and (C) 315 cm^{-1} and 331 cm^{-1} are assigned as NH_2 rocking vibration and C-S-Au angle bending vibration - representative of two products viz., p-mercaptobenzamide and p-mercaptobenzonitrile, respectively. (633 nm; laser power: 2.7 mW, acquisition time: 0.5 s)

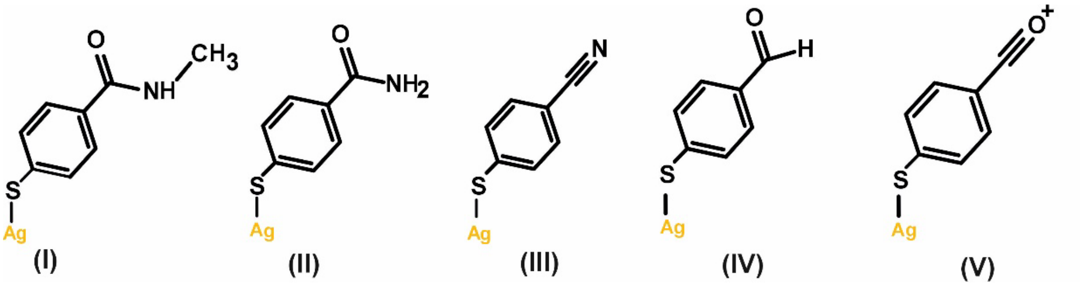

Supplementary Figure 2. Molecular structure of Ag bound different plausible reaction products: (I) N-methyl-4-sulfanylbenzamide (NMSB) – starting material, and plausible reaction products (II) Ag-S(C_6H_4)$CONH_2$, (III) Ag-S(C6H4)CN, (IV) Ag-S(C_6H_4)CHO, and (V) Ag-S(C_6H_4)CO^+.

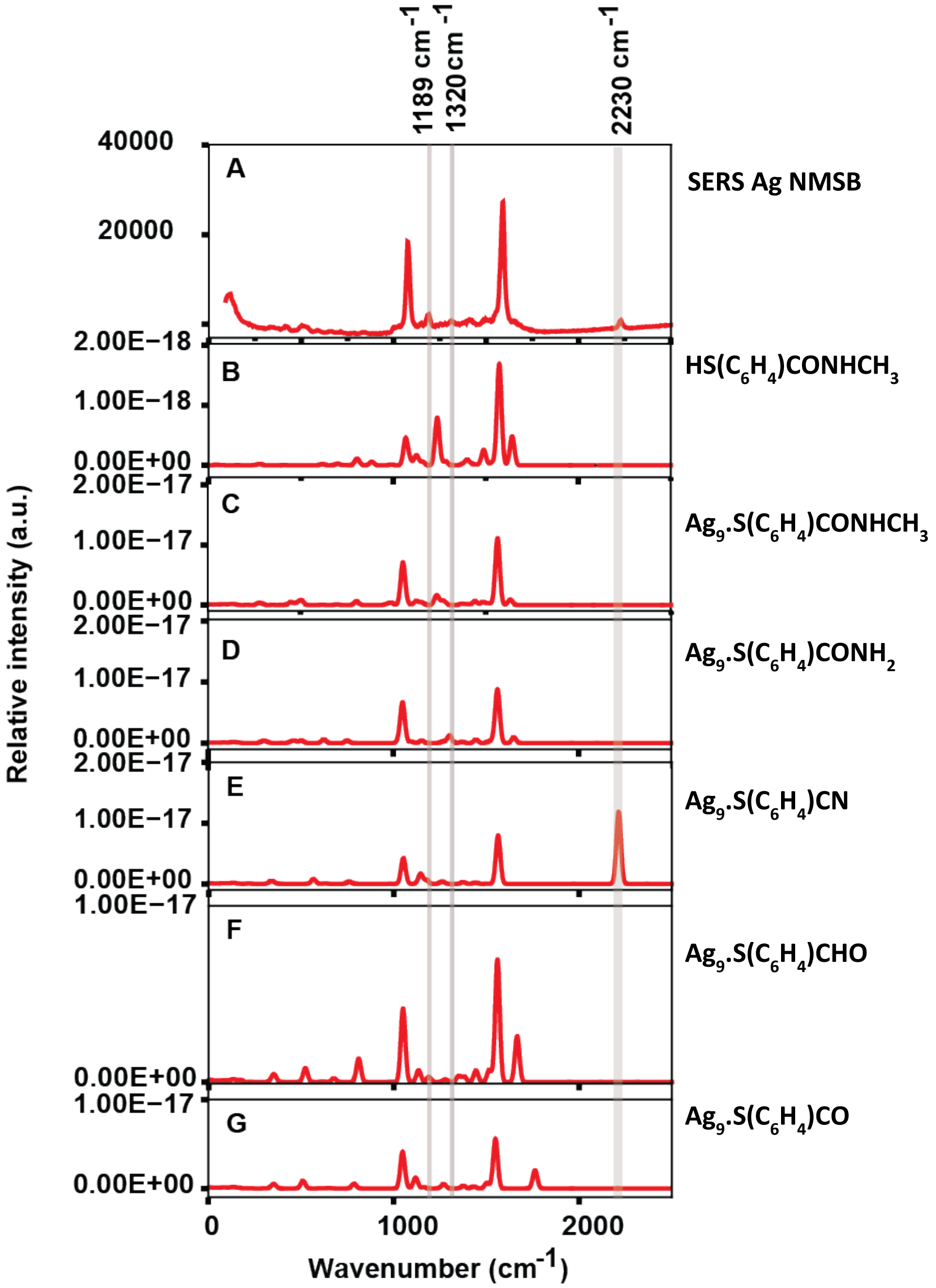

Supplementary Figure 3. Comparison of SERS spectra of NMSB treated Ag after laser irradiation and calculated Raman spectra of plausible reaction products: (A) SERS spectra obtained after irradiation of NMSB coated Ag aggregates with 633 nm laser; (B-G) Raman spectra of plausible product molecule calculated at the B3LYP/aug-cc-pVDZ level with scaling of 0.96, Gaussian broadening with 30 cm^{-1} FWHM. Calculated spectra correspond to various relevant molecules attached to Ag_9 clusters considering the Ag-S mode of bonding. A relative red-shift in the SERS spectra could be seen (for the marked peak) when compared to the calculated Raman spectra in most cases.

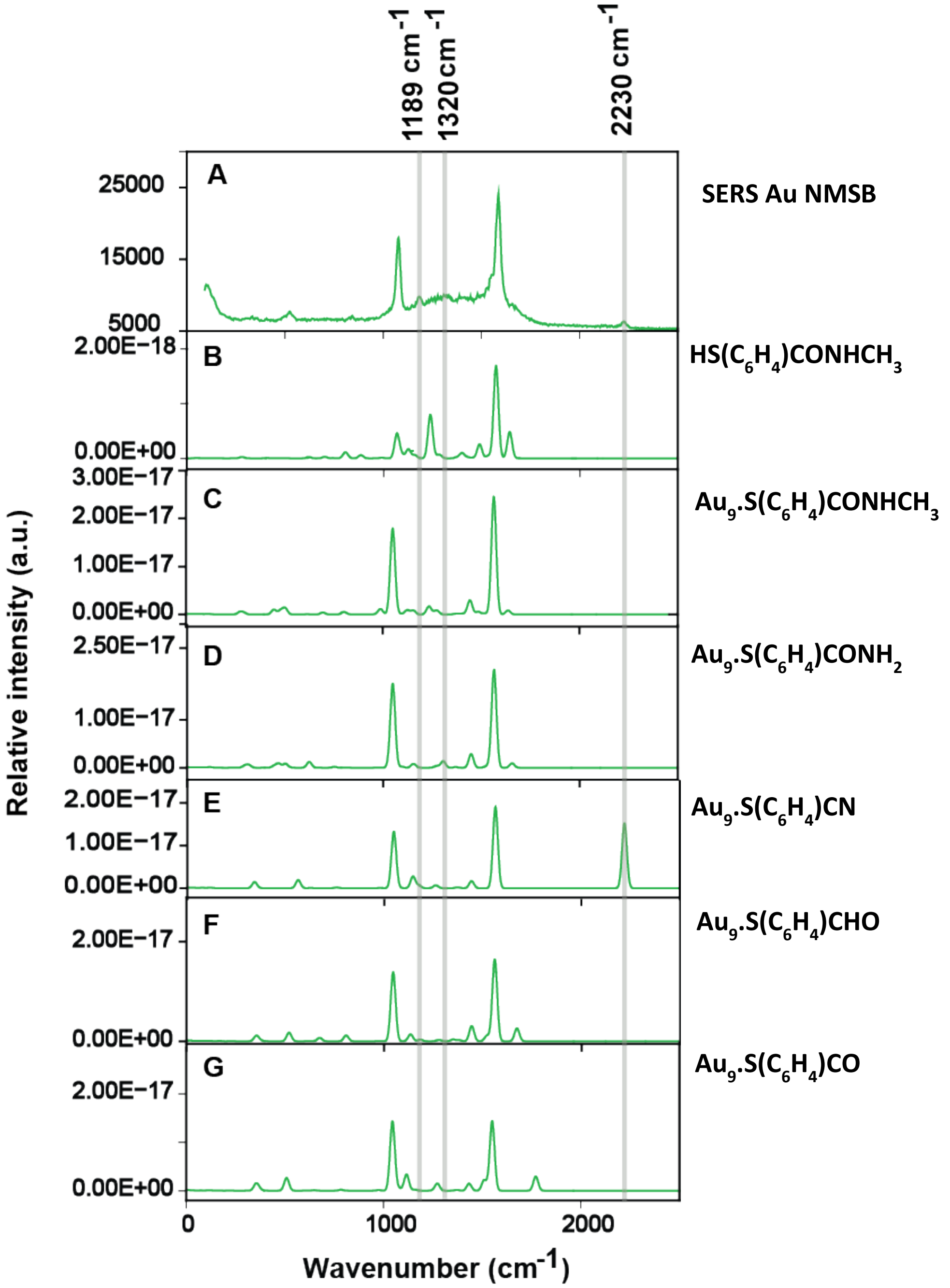

Supplementary Figure 4. Comparison of SERS spectra of NMSB treated Au after laser irradiation and calculated Raman spectra of plausible reaction products: (A) SERS spectra obtained after irradiation of NMSB coated Au aggregates with 633 nm laser; (B-G) Raman spectra of plausible product molecule calculated at the B3LYP/aug-cc-pVDZ level with scaling of 0.96, Gaussian broadening with 30 cm^{-1} FWHM. Calculated spectra correspond to various relevant molecules attached to Au_9 clusters considering the Au-S mode of bonding.

Supplementary Discussion 1.

DEA discussion:

Supplementary Table 1 summarizes the peak positions and the respective thresholds. Upon electron attachment to the MCB molecule, four fragment anions are observed. At low energies, hydrogen atom dissociation is observed, $[M–H]^-$, at *m*/*z* 166. The peak at low energies is considerably asymmetric, with a maximum at 1.1 eV, another peak is observed at 4.4 eV. The ion yield of $[M–H]^-$ is by two orders of magnitude higher compared to all other yields. The ion at *m*/*z* 109, $[M–C_2H_4NO]^-$, is observed at energies above 4.3 eV, with two peaks maxima at 5.7 eV and 8.6 eV. Finally, SH^- and S^- ions are observed at *m*/*z* 33 and 32, respectively. Both ions have very similar yield curves with two peaks at higher electron energies, suggesting they share the same temporary negative ion state and are produced within a similar pathway. The ratio of the first and the second peak is ~0.6 in the SH^- ion yield and decreases to ~0.4 for S^-.

The neutral MCB molecule is calculated to be almost planar, with the S–H bond lying in the plane of the benzene ring, the cis/trans orientations of the SH group have almost the same energy (difference below 0.01 eV). Our calculations predict that both vertical and adiabatic electron affinities are close to zero eV. Using the *ω*B97XD/aug-cc-pVTZ(H)+ and B3LYP/aug-cc-pVTZ(H)+ methods with an enlarged basis set, the vertical electron affinity of 0.01 eV and 0.23 eV is obtained, respectively; the adiabatic electron affinity is calculated as 0.43 eV, 0.28 eV and 0.22 eV at B3LYP/aug-cc-pVDZ, *ω*B97XD/aug-cc-pVDZ and CCSD(T)/aug-cc-pVDZ//*ω*B97XD/aug-cc-pVDZ levels, respectively. In the minimum structure of the anion, the additional electron is positioned in a delocalized π* orbital, with the S–H group perpendicular to the benzene ring shown in Supplementary Figure 5.

Suggested dissociation pathways are summarized in Supplementary Figure 5. Dissociation of a hydrogen atom is calculated to require 0.64 eV for the least bound hydrogen of the S–H bond, other hydrogen atoms require at least 0.8 eV more for dissociation. The experimental threshold of the asymmetric peak with its vertical onset is 0.67 eV, in very good agreement with the calculated threshold. We also found ion yield with very low intensity below the vertical onset, which we ascribe to an impurity. Apart from the computational uncertainty, the presence of the signal at low energies may be also explained by the average thermal energy of 0.31 eV and 0.41 eV at 298 K and 353 K, respectively. The predicted direct dissociation is in agreement with the high intensity of the main peak in the experimental spectrum.

The $[M–C_2H_4NO]^–$ ion is predicted to emerge through direct dissociation of the CH_3NHCO group, with a dissociation energy of 2.93 eV, well below the experimental threshold. The $SH^–$ and $S^–$ ions could be formed through pre-dissociation of the SH group over a barrier of 1.05 eV reflected in Supplementary Figure 5. Then, the whole $SH^–$ moiety might leave with the total reaction energy of 1.45 eV, or the hydrogen atom might re-attach to the benzene cycle, forming $S^–$, with the reaction energy of 0.43 eV. The calculated dissociation energies lie below the experimental thresholds. Alternatively, the $S^–$ ion might be also formed in the second step from the dissociated $SH^–$ ion, this requires however more than 5 eV of energy, and this channel should be thus active only at higher electron energies in the experiment.

Supplementary Table 1: Summary of anions observed upon electron attachment to isolated NMSB, along with their mass, suggested molecular structure, peak positions, and experimental thresholds. Calculated thresholds are given at the CCSD(T)/aug-cc-pVDZ//ωB97XD/aug-cc-pVDZ level of theory.

Mass (u)	Anion	Peak Positions (eV)		Threshold (eV)	
		1	2	Exp.	Theory
166	$[M–H]^–$	1.1	4.4	0.67	0.64
109	$[M–C_2H_4NO]^–$	5.7	8.6	4.3	2.93
33	$SH^–$	5.1	7.4	2.5	1.45
32	$S^–$	4.9	7.2	3.6	0.43

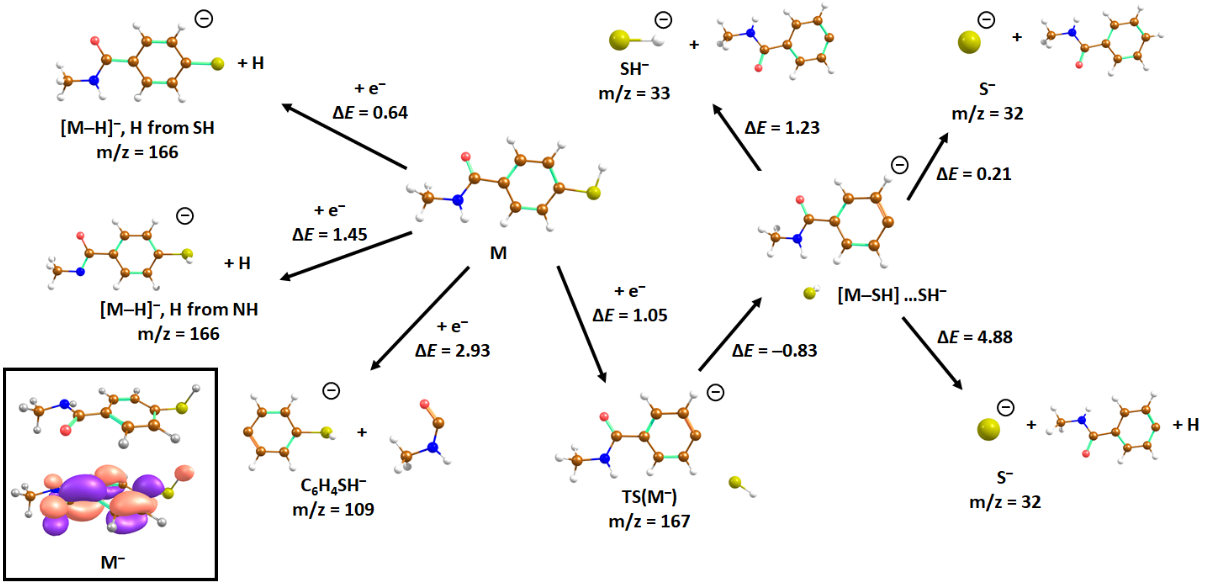

Supplementary Figure 5. Possible reaction route and DFT calculated reaction energies: Suggested dissociation pathways and reaction energies (in eV) as calculated at the CCSD(T)/aug-cc-pVDZ//*ω*B97XD/aug-cc-pVDZ level. In the frame, the minimum structure

of M⁻ is given, along with the orbital occupied by a single electron as calculated at the ωB97XD/aug-cc-pVTZ(H)+ level. (Refer to Supplementary data 1)

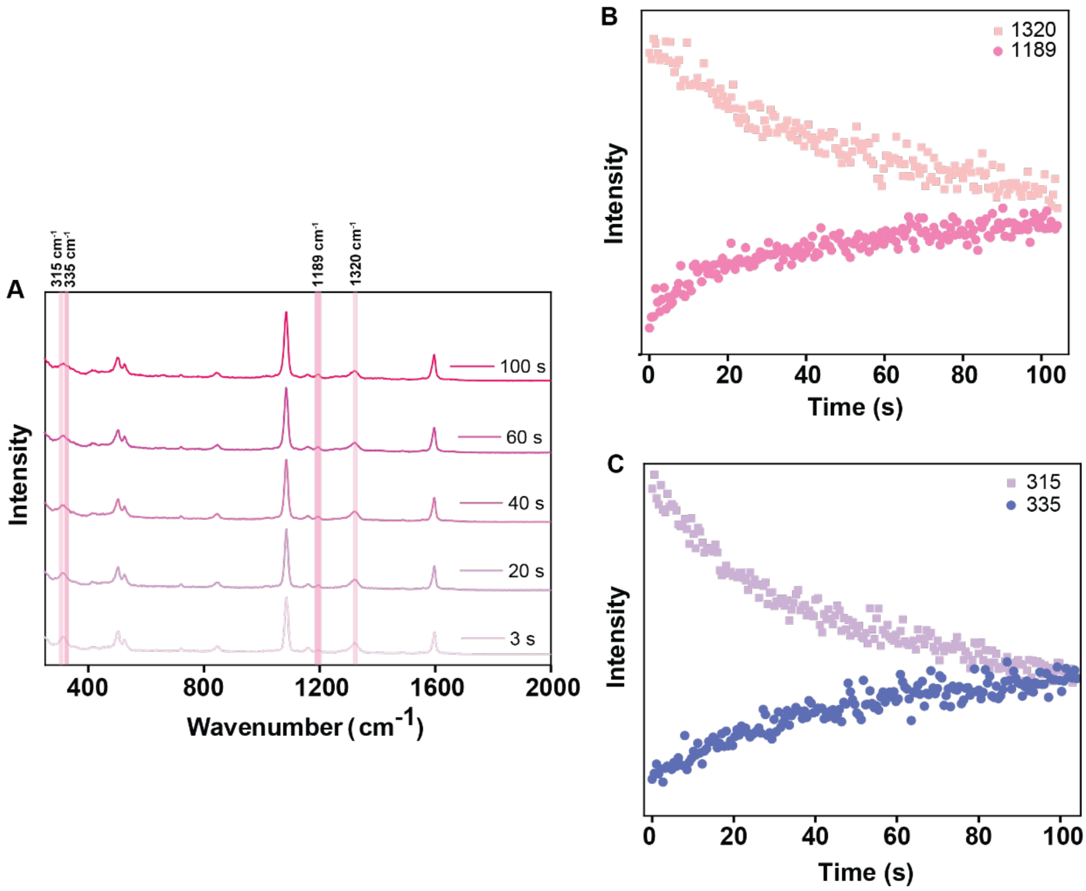

Supplementary Figure 6. Time evolution of the transformation of a secondary amide on Ag aggregates tracked by SERS with 785 nm laser: Representative SERS spectra obtained at different time intervals of the chemical transformation of NMSB on Ag-aggregates under 785 nm laser excitation are shown in (A). The time trajectories of the peaks at (B) 1320 cm^{-1}, and 1189 cm^{-1} correspond to the loss of the amide III band, and the appearance of the peak due to primary amide respectively, and (C) 315 cm^{-1} and 335 cm^{-1} are assigned to NH_2 rocking vibration and C-S-Ag angle bending vibration - representative of two products viz., p-meracptobenzamide and p-mercaptobenzonitrile respectively. (785 nm; laser power: 2 mW, acquisition time: 0.5 s).

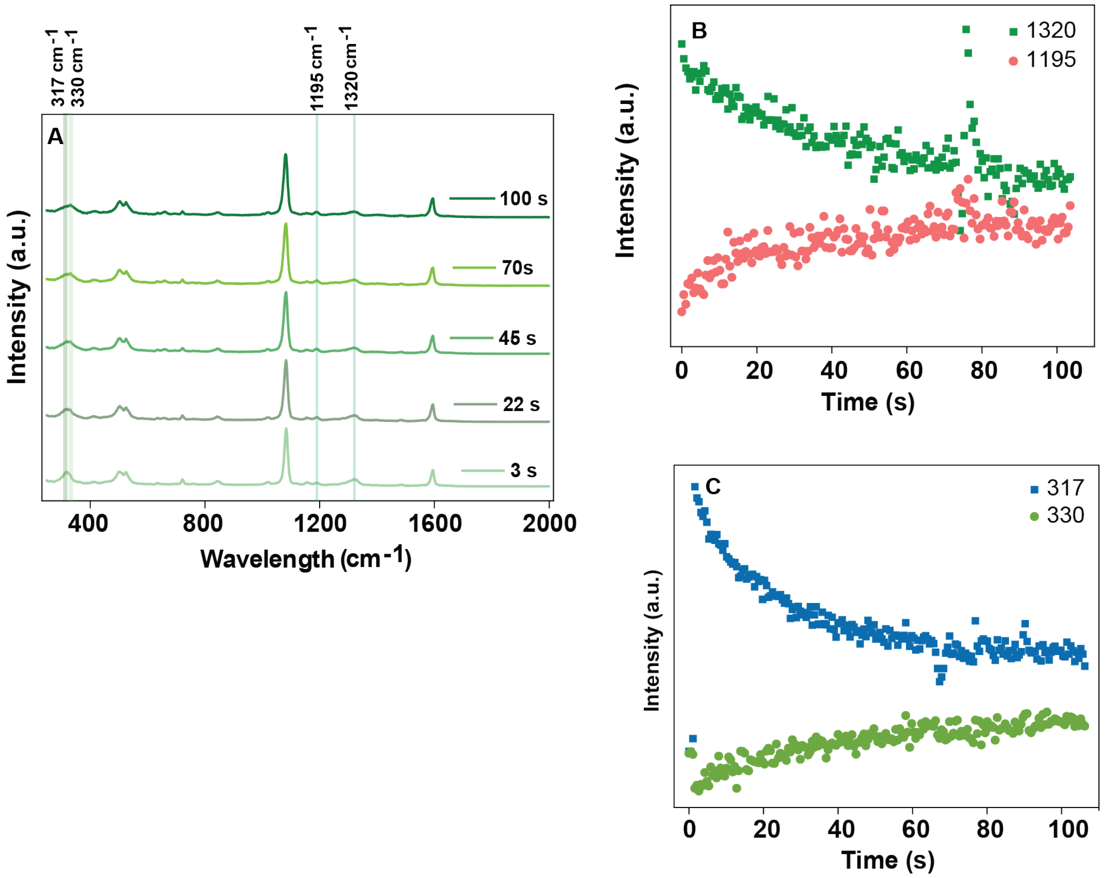

Supplementary Figure 7. Time evolution of the transformation of a secondary amide on Au aggregates tracked by SERS with 785 nm laser: Representative SERS spectra obtained at different time intervals of the chemical transformation of NMSB on Au-aggregates under 785 nm laser excitation are shown in (A). The time trajectories of the peaks at (B) 1320 cm^{-1}, and 1195 cm^{-1} correspond to the loss of the amide III band, and the appearance of the peak due to primary amide respectively, and (C) 317cm^{-1} and 330 cm^{-1} are assigned to NH_2 rocking vibration and C-S-Ag angle bending vibration - representative of two products viz., p-meracptobenzamide and p-mercaptobenzonitrile respectively. (785 nm; laser power: 3 mW, acquisition time: 0.5 s).

Supplementary Discussion 2.

Kinetic Fitting and Reaction Rate Constant

We consider the following general equation (Supplementary Equation 1) for the dissociative conversion of NMSB at the plasmonic nanocavity:

$$\mathrm{NMSB} + e^{-} \rightleftharpoons \mathrm{NMSB}^{\#-} \xrightarrow{\mathrm{H}^{+}} \mathrm{MBAm} + \mathrm{CH_3} \longrightarrow \mathrm{MBN} + \mathrm{CH_3} + \mathrm{H_2O}$$

(Supplemetary Equation 1)

The equation above describes the hot-electron-induced dissociation of the NMSB molecule in the presence of a proton source to form MBAm and a methyl radical as a byproduct. The $MBAm$ further transforms to generate MBN where, $MBAm$ = p-mercaptobenzamide, MBN = p-mercaptobenzonitrile, and CH_3 = methyl radical.

A general rate equation of the above reaction can be written as follows:

$$\text{Rate} = k \cdot [\text{NMSB}]\ [\text{e}^-] \qquad \text{(Supplemetary equation 2)}$$

Considering fast excitation and relaxation of hot-charge carriers under continuous wave (CW) illumination, the time-average concentration of the hot electrons is considered constant which allows us to consider the process to follow a pseudo-first-order rate law.[1]

Therefore, the rate equation can be written as,

$$\text{Rate} = k_1 \cdot [\text{NMSB}]\text{, where } k_1 = k \cdot [\text{e}^-] \qquad \text{(Supplemetary equation 3)}$$

$$\text{Rate} = \frac{-\,\text{d}[\text{NMSB}]}{\text{d}t} = k_1 \cdot [\text{NMSB}] \qquad \text{(Supplemetary equation 4)}$$

We take into account a fractal kinetic term in the rate equation to include the inhomogeneity of the substrate. (find similar discussion in ref 12) The inhomogeneous distribution of the reaction site induces a time dependence of the electron transfer and hence the rate constant, which is taken care of by considering the fractal term expressed by Supplementary equation 5.[2]

$$k_1 = k_f \cdot t^{-h}\ ,\ 0 \leq h \leq 1 \text{ and } t \geq 1 \qquad \text{(Supplemetary equation 5)}$$

where k_1 refers to the time-dependent reaction rate constant, k_f is the kinetic corrected time-independent rate constant will be used in the kinetic analysis to deduce the reaction rate constant and h represents the kinetic fractal term.[3]

We substitute Supplementary Equation 5 in Supplementary Equation 4, to obtain the below expression:

$$\text{Rate} = \frac{-\text{d}[\text{NMSB}]}{\text{d}t} = k_1 \cdot [\text{NMSB}] = k_f\ t^{-h} \cdot [\text{NMSB}] \qquad \text{(Supplemetary equation 6)}$$

Integrating Supplementary Equation 6 leads to the first-order fractal Supplementary Equation 7,

$$\int_{[\text{NMSB}]_0}^{[\text{NMSB}]} \frac{\text{d}[\text{NMSB}]}{[\text{NMSB}]} = \int_0^t k_f \cdot t^{-h}.\,\text{dt}$$

$$\Rightarrow \ln\frac{[\text{NMSB}]_0}{[\text{NMSB}]} = \frac{k_f}{1-h} \cdot t^{(1-h)} \qquad \text{(Supplemetary equation 7)}$$

We consider the extracted time trace SERS intensity to represent the concentration at time "t" of the reaction. For example, the reaction time trace for the peak corresponding to 1320 cm^{-1} is fitted into Supplementary equation 7 to deduce the reaction rate constant given by, k_f under different reaction conditions. Supplementary equation 7 represents a power law function represented by $y = a \cdot t^b$ and therefore $k_f = (1 - h) \cdot a = b \cdot a$, where $b = (1 - h)$.

A representation of the kinetic fit using Supplementary equation 7, under 633 nm excitation for the reaction of NMSB on the Ag surface is shown in Supplementary Figure 8.

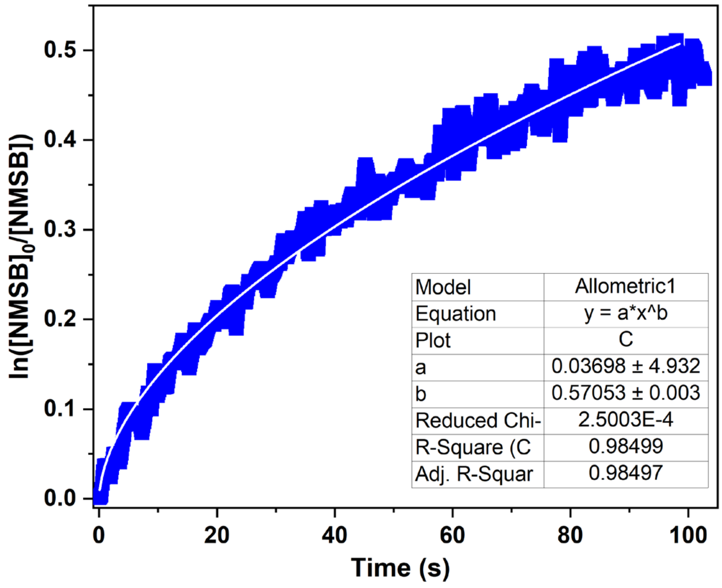

Supplementary Figure 8. Reaction rate versus time plot: $\ln \frac{[\mathrm{NMSB}]_0}{[\mathrm{NMSB}]}$ versus time plot when fitted to first order (equation S7) integrated rate expression. Pale yellow curve showing the 2corresponding fit (Laser: 633 nm; 0.5 mW).

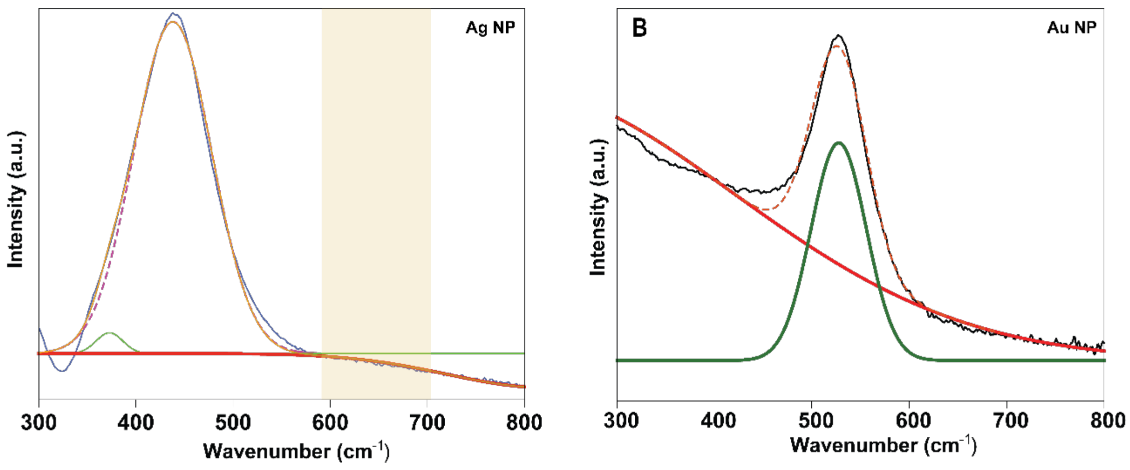

Supplementary Figure 9. Deconvolution of UV-vis spectra of NMSB coated Ag and Au dispersion. Deconvoluted UV-vis spectrum of NMSB treated (A) AgNPs after centrifugal wash. Blue curve – original spectrum, red dashed curve – cumulative fit, orange curve – fit 1, green curve – fit 2, red curve -fit 3. Fit 3 shows a secondary plasmon band in the 600-700 nm range. (B) AuNPs after centrifugal wash. Black curve – original spectrum, red dashed curve – cumulative fit, green curve – fit 1, red curve – fit 2. No secondary plasmon band was observed for the NMSB-treated AuNP in the solution phase.

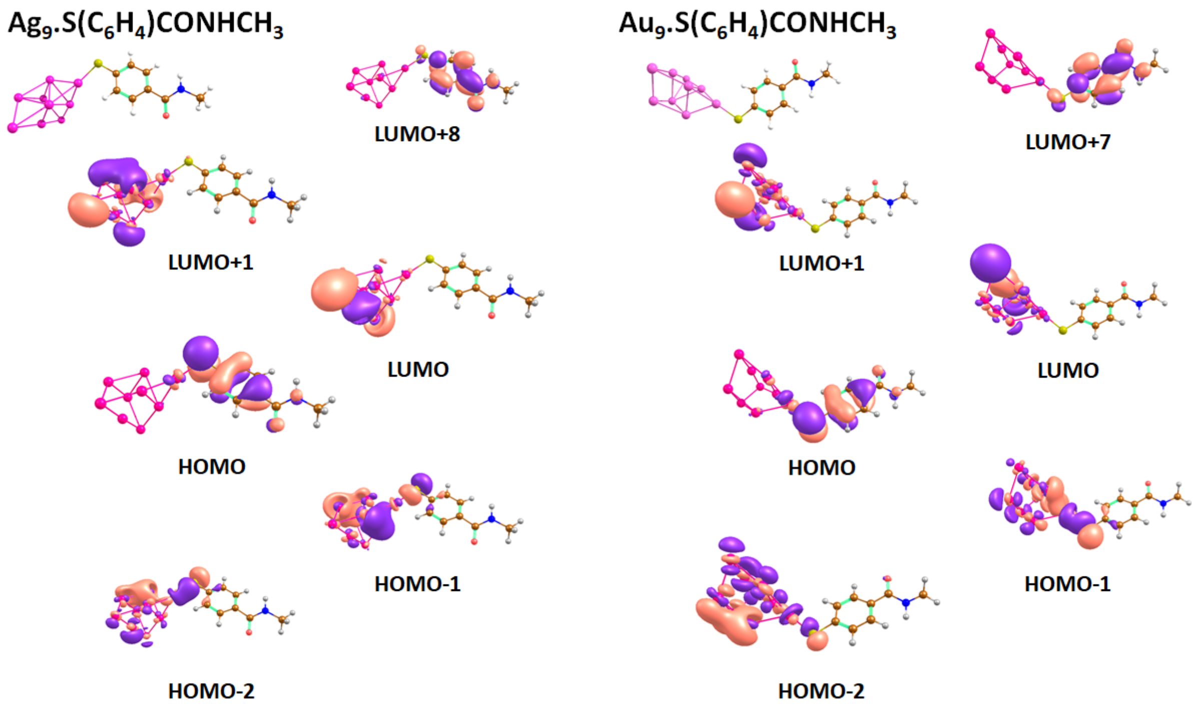

Supplementary Figure 10. DFT calculated and optimized molecular structure and orbitals of NMSB: The optimized structures and the molecular orbitals of the NMSB molecule adsorbed on an Ag atom and Au atom in a M_9 cluster, using B3LYP/aug-cc-pVDZ. LUMO+8 and LUMO+7 are the first virtual orbitals that are localized on the adsorbed molecule in the Ag/NMSB and Au/NMSB clusters, respectively. (Refer to Supplementary data 1)

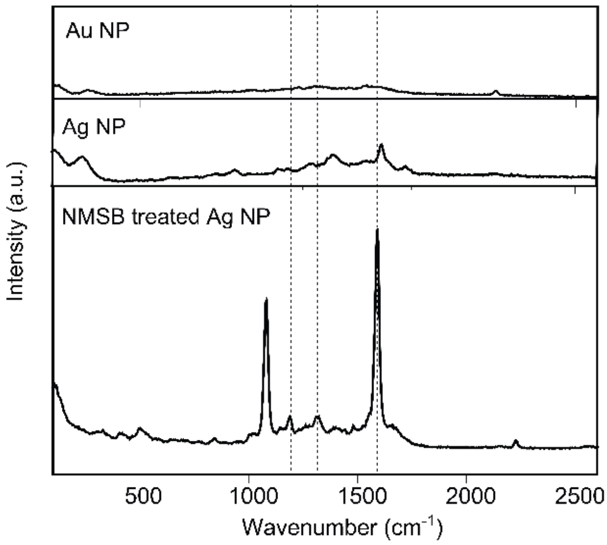

Supplementary Figure 11. SERS spectra of bare NPs and NMSB treated NPs: SERS spectra recorded on bare aggregated 40 nm citrate capped Au NPs and Ag NPs (633nm laser, laser power - 500uW, acquisition time - 50 ms). The SERS spectrum of NMSB-coated Ag NPs is shown for reference.

Supplementary References

1. Zhang, Z., Zhang, C., Zheng, H., Xu, H. Plasmon-Driven Catalysis on Molecules and Nanomaterials. *Acc. Chem. Res.* **52**, 2506–2515 (2019).
2. Kopelman, R. Fractal Reaction Kinetics. *Science* **241**, 1620–1626 (1988).
3. Dutta, A. et al. Kinetics and mechanism of plasmon-driven dehalogenation reaction of brominated purine nucleobases on ag and au. *ACS Catal* **11**, 8370–8381 (2021).
